# Real-world impact of nirsevimab on RSV hospitalizations in children under 2 years of age: retrospective monocentric study in a large German tertiary pedatric center

**DOI:** 10.1186/s40348-026-00232-5

**Published:** 2026-05-07

**Authors:** Moritz Bauer, Anke Wendt, Marcus A. Mall, Horst von Bernuth, Stephanie Thee

**Affiliations:** 1 Department of Pediatric Respiratory Medicine, Immunology and Critical Care Medicine, Charité-Universitätsmedizin, Berlin, Corporate Member of Free University and Humboldt University and Berlin Institute of Health, Mittelallee 8, Berlin, Germany; 2 German Centre for Child and Adolescent Health (DZKJ), partner site Berlin, Berlin, Germany; 3 German Centre for Lung Research (DZL) , associated partner, Berlin, Germany; 4 Cluster of Excellence ImmunoPreCept, Charité - Universitätsmedizin Berlin, Berlin, Germany; 5 Labor Berlin Charité-Vivantes, Department of immunology, Berlin, Germany; 6 Charité - Universitätsmedizin Berlin, corporate member of Freie Universität Berlin, Humboldt-Universität zu Berlin, and Berlin Institute of Health (BIH), Berlin-Brandenburg Center for Regenerative Therapies (BCRT), Berlin, Germany; 7 Berlin Institute of Health at Charité - Universitätsmedizin Berlin, Berlin, Germany

**Keywords:** Respiratory syncytial virus, Nirsevimab, Passive immunization

## Abstract

**Background:**

Respiratory syncytial virus (RSV) infection is a leading cause of hospitalization among infants and young children. Broad immunization with the monoclonal antibody nirsevimab represents a promising preventive strategy. However, real-world data on its impact at the population level remain limited, particularly in routine clinical settings.

**Methods:**

This retrospective single-center study evaluated a cohort of 261 children under two years of age hospitalized with RSV across three seasons (2018–2019, 2023–2024, 2024–2025) at a Berlin tertiary care hospital.

**Results:**

We analyzed admissions and clinical courses of RSV-associated hospitalizations in children < 2 years of age at a large tertiary care center in Germany between 2018 and 2025. Following the nationwide introduction of nirsevimab in 2024, the number of RSV-related hospitalizations in our center decreased by 51% compared to the previous season. During the 2024/2025 season 90.2% of hospitalized patients had not received nirsevimab immunization.

**Conclusion:**

The introduction of nirsevimab was associated with a substantial reduction in RSV-related hospitalizations in infants and young children in a real-world clinical setting. These findings support a beneficial role of passive immunization with nirsevimab and highlight its potential to reduce RSV-related disease burden.

## Background

RSV is a major cause of mortality, morbidity and recurrent hospitalizations among infants and young children globally [1]. With almost 100,000 hospitalizations between 2019 and 2022 [2], RSV imposes a significant burden on the German pediatric healthcare system, particularly during seasonal epidemics, highlighting the urgent need for effective preventive strategies. One strategy involves passive immunization with the monoclonal antibody nirsevimab, which targets the prefusion F protein of RSV and has an extended half-life of more than 60 days, allowing a single dose to protect for an entire season. This half-life is substantially longer than the approximately 30-day half-life of palivizumab, a humanized monoclonal antibody long used for RSV immunization in high-risk infants but requiring monthly dosing due to its shorter durability [3, 4]. Nirsevimab received EU approval in 2022 and has since been adopted in several countries. In 2024, the German Standing Committee on Vaccination (STIKO) at the Robert Koch Institute introduced a systematic recommendation for nirsevimab immunization in all children under 1 year of age, and in children under 2 years of age with comorbidities that predispose to a severe course of RSV infection, with immunization costs fully covered through the standard healthcare system [5]. Thus, the 2024/2025 season constitutes the first season after the introduction of this nationwide passive immunization strategy.

Early surveillance data from the Robert Koch Institute and the Federal Ministry of Health suggest a marked decline in RSV incidence and hospitalizations during the 2024/2025 season compared to the preceding year, with a 55% reduction in hospital admissions among infants under 1 year of age [6]. Although these early findings are promising, individual-level data remain limited, and the proportion of immunized children among hospitalized patients has not yet been fully documented. Against this background, we conducted a retrospective analysis of the clinical courses of RSV-related diseases in children under 2 years of age at a large tertiary pediatric center in Berlin, aiming to assess the real-world impact of the introduction of nirsevimab.

## Methods

### Study design

In this single-center retrospective study pediatric patients under 2 years of age who were diagnosed with RSV and consequently hospitalized at the Children’s Hospital of Charité - Universitätsklinikum Berlin were included. We enrolled a total of 261 children who were hospitalized during three RSV seasons: 2018–2019 (pre-COVID-19), 2023–2024 (pre-nirsevimab), and 2024–2025 (post broad recommendation of nirsevimab). We defined the period from October to May as the RSV season for children in Germany, as epidemiological surveillance data consistently indicate that RSV activity and pediatric hospitalizations peak during these months, reflecting the virus’s typical cold-season circulation pattern in temperate climates.

### Study population and data collection

Eligible patients were identified through the hospital’s electronic medical records using ICD-10 codes for RSV infection (J12.1, J20.5, J21.0). The primary outcome of this study was hospitalization due to laboratory-confirmed RSV infection (PCR or antigen test) in children aged < 2 years of age. RSV infection was diagnosed as part of routine clinical care, with testing performed in all children hospitalized for respiratory symptoms. ICD-10 codes were used solely to identify potential cases for review, and cases without a positive PCR or antigen test were excluded. The choice of diagnostic method depended on clinical practice at the time of presentation and was not standardized within the study protocol. Co-infections with other respiratory viruses were not systematically assessed, as multiplex PCR testing was performed at the discretion of the treating physicians. While most children required supplemental oxygen due to respiratory distress, others were admitted for alternative clinical reasons, including dehydration, feeding difficulties, or the need for observation due to fever or general clinical status. Patients with missing diagnostic confirmation or incomplete clinical data were excluded. Demographic, clinical, and laboratory data were extracted from the electronic medical records. Collected variables included age, sex, gestational age, immunization status, comorbidities (e.g., prematurity, cyanotic heart disease, chronic lung disease), length of hospital stay and need for oxygen therapy or mechanical ventilation. Because some patients required escalation or changes in respiratory support during hospitalization, individual patients may be represented in more than one category; therefore, categories are not mutually exclusive. Respiratory support followed routine clinical practice: supplemental oxygen for SpO₂ <90% and HFNC for persistent respiratory distress under conventional oxygen, with escalation left to clinical judgment.

Clinical symptoms were documented as part of routine clinical care without the use of a standardized assessment tool, and analyses were limited to symptoms that were consistently recorded across the cohort. Immunization status was assessed through review of the medical records and was primarily based on parental report at admission; where available, this information was verified using written documentation in the child’s immunization record.

### Statistical analysis

Statistical analysis was carried out using GraphPad Prism, V.9.1.1 for macOS. To summarize patient characteristics descriptive statistics were used. Categorical variables were presented as counts and percentages. Continuous variables were expressed as median with interquartile range (IQR). Because population-level denominators were not available, analyses were restricted to descriptive comparisons of hospitalization counts between seasons. Hospitalization count ratios were calculated for seasonal comparisons, and approximate 95% confidence intervals were derived assuming Poisson-distributed counts. These estimates were intended to describe the magnitude and precision of observed differences rather than to support formal hypothesis testing.

## Results

A total of 340 patients were hospitalized with RSV during the observation period. However, 79 patients were excluded from the final analysis due to incomplete data. Missingness in these cases frequently affected multiple key variables simultaneously (including demographic and clinical parameters) rather than isolated data points, precluding a systematic comparison between included and excluded patients. Infection profiles and corresponding patient characteristics of the 261 patients included in the analysis are summarized in Table 1. Males accounted for a slight majority in all seasons (overall 58.2%). Typical clinical symptoms at presentation included cough (78.5%) and tachypnea (67.8%), with apnea reported in 1.1% of patients. After implementation of the national recommendation for nirsevimab, RSV-related hospitalizations in our cohort decreased markedly compared to both the 2018/2019 season (hospitalization count ratio 0.56, 95% CI 0.40–0.78) and the 2023/2024 season (count ratio 0.49, 95% CI 0.35–0.68), corresponding to approximate reductions of 44% and 51%, respectively. Notably, more than 95% of all patients hospitalized throughout the observation period had not received prior RSV immunization. During the 2024/2025 season, the immunization rate among eligible children was 7.4%. None of the patients older than 12 months who were eligible based on comorbidities (*n* = 3) had received nirsevimab. In the cohort of hospitalized patients, prematurity was the most frequent risk factor, occurring in 15.3% of cases, followed by chronic lung disease. The prevalence of underlying comorbidities remained stable over the entire observation period. Across all seasons, 74,3% of patients required oxygen supplementation, most commonly via nasal oxygen (65,1%) or high-flow therapy (24,1%). Invasive ventilation was no longer required in any child after the 2018/2019 season, and no deaths were recorded throughout the entire study.

**Table 1:** Overview of patient characteristics (*n*= 261)

	Total		2018-2019		2023-2024		2024-2025	
Patients, *n* (%)	261	(100%)	97	(37.2%)	110	(42.1%)	54	(20.7%)
Sex male, n (%)	152	(58.2%)	50	(51.5%)	68	(61.8%)	34	(63%)
Sex female, n (%)	109	(41.8%)	47	(48.5%)	42	(38.2%)	20	(38%)
Age 0–12 months, n (%)	193	(73.9%)	26	(26.8%)	24	(21.8%)	18	(33.3%)
Age, months^1^	4	(1–13)	4	(1–13)	4	(2–11)	5	(3–14)
Symptoms at presentation								
Tachypnea n (%)	177	(67.8%)	58	(59.8%)	86	(78.2%)	33	(61.1%)
Cough, n (%)	205	(78.5%)	70	(72.2%)	88	(79.3%)	47	(87%)
Apnea, n (%)	3	(1.1%)	2	(2.1%)	0	(0%)	1	(1.9%)
Immunization status								
Immunization Palivizumab, n (%)	8	(3.1%)	6	(6.2%)	2	(1.8%)	0	(0%)
Immunization Nirsevimab, n (%)	4	(1.5%)	0	(0%)	0	(0%)	4	(7.4%)
Comorbidities								
Gestational age < 37 weeks, n (%)	23	(8.8%)	10	(10.3%)	6	(4.5%)	7	(13%)
Gestational age < 32 weeks, n (%)	17	(6.5%)	10	(10.3%)	4	(3.6%)	3	(5.6%)
Chronic lung disease, n (%)	15	(5.7%)	6	(6.2%)	6	(5.5%)	3	(5.6%)
Cyanotic heart disease, n (%)	10	(3.8%)	5	(5.1%)	3	(2.7%)	2	(3.7%)
Neuromuscular disease, n (%)	5	(1.9%)	1	(1%)	1	(0.9%)	3	(5.5%)
Malignant disease, n (%)	1	(0.4%)	1	(1%)	0	(0%)	0	(0%)
Congenital immunodeficiency, n (%)	6	(2.3%)	2	(2.1%)	2	(1.8%)	2	(3.7%)
Iatrogenic immunodeficiency, n (%)	1	(0.4%)	1	(1%)	0	(0%)	0	(0%)
Hospital course								
Hospital stay, days^1^	5	(3–8)	6	(4–8)	5	(3–7)	5	(3–8)
Respiratory support total^2^, n (%)	194	(74.3%)	73	(75.3%)	82	(75%)	41	(76%)
Nasal mask, n (%)	170	(65.1%)	51	(52.6%)	79	(71.1%)	40	(74%)
High-flow therapy, n (%)	63	(24.1%)	32	(33%)	20	(6.4%)	11	(20.3%)
Non-invasive ventilation, n (%)	22	(8.4%)	12	(12.4%)	7	(18.2%)	3	(5.6%)
Invasive ventilation, n (%)	4	(1.5%)	4	(4.1%)	0	(0%)	0	(0%)
Respiratory support unvaccinated, n (%)	188	(75.5%)	68	(74.7%)	81	(75%)	39	(78%)
Respiratory support vaccinated, n (%)	9	(75%)	5	(83%)	2	(100%)	2	(50%)

^1^ Reported in median (IQR)

^2^ Due to SpO₂ < 90%

## Discussion

We present real-world data from a large German tertiary care hospital demonstrating a substantial reduction in RSV-related hospitalizations following the nationwide introduction of nirsevimab, along with a detailed characterization of hospitalized patients, the majority of whom were not immunized.

Our center accounted for 33% of RSV-related hospitalizations in this age group within the Berlin metropolitan region during the 2023/2024 season and 25.6% during the 2024/2025 season [7], with an overall 51% reduction in RSV-related admissions to our hospital after the nationwide nirsevimab recommendation. Notably, the local reduction closely mirrors recently published national data from the Robert Koch Institute, which reported an approximate 55% decrease in RSV-related hospitalizations after nirsevimab rollout [6]. One plausible explanation for the lower reduction in RSV hospitalizations observed in our cohort, compared to other European countries, is the considerably higher real-world uptake of nirsevimab in those regions. For example, national observational data from Italy report coverage of around 90% with reductions in RSV-related hospitalizations of up to ~ 80–86% following universal implementation [8], highlighting how high coverage drives greater population-level impact, which may not be fully mirrored in our setting [9]. Given that the prevalence of high-risk comorbidities and median age in our cohort remained similar across seasons, the observed reduction in hospitalizations is likely attributable to immunization itself rather than shifts in patient risk profiles.

This study has several limitations. First, the recently revised national RSV immunization strategy primarily targets children aged ≤ 12 months, which rendered 31.5% of our patients ineligible for RSV immunization based on age alone. We nevertheless included children aged < 24 months, as nirsevimab is approved for use in this age group when significant risk factors are present. Second, natural fluctuations in RSV circulation and the non-randomized, single-center study design substantially limit any causal interpretation of the observed differences. In our study population, RSV activity largely followed the expected seasonal pattern during the analyzed seasons, and no pronounced off-season peak was observed in the post-pandemic period in Berlin [10]. However, our analysis relies on absolute numbers of hospitalizations without a defined population denominator, and is therefore inherently susceptible to external influences unrelated to immunization. In particular, changes in referral patterns, healthcare utilization and admission thresholds over time may have influenced both case ascertainment and hospitalization rates.

These factors limit the comparability between seasons and preclude attributing observed reductions in hospitalizations directly to the introduction of nirsevimab. In addition, as this study was conducted at a large tertiary care center, the findings may not be generalizable to other healthcare settings, particularly smaller hospitals or rural regions.

Finally, the study period encompassed only the first full season following the nationwide rollout of nirsevimab, resulting in a limited number of immunized children and restricting our analysis to descriptive statistics. As no data on overall immunization coverage in the underlying population were available, the observed low proportion of immunized patients among hospitalized cases cannot be interpreted in terms of effectiveness. Given the retrospective, monocentric design and the lack of a population denominator, our study does not allow estimation of vaccine effectiveness or population-level impact.

## Conclusions

The reduction in RSV-associated hospitalizations observed in our cohort provides important real-world evidence supporting the effectiveness of nirsevimab and underscores its potential to optimize RSV prevention strategies. Expanding immunization coverage therefore remains a key public health priority. Future studies should investigate long-term immunity after passive immunization, particularly whether attenuated infection or disease provides the anticipated protective immune response. Recently published data demonstrating protection lasting up to six months suggest that the duration of immunity is longer than previously assumed [11]. Consequently, the immunization window could be substantially extended, which, given the very limited timeframe during the 2024/2025 season, may markedly increase immunization rates. Continued surveillance across subsequent seasons will be essential to validate these trends and to refine immunization strategies to maximize population-level benefits. Building on these findings, it is also important to consider maternal immunization as a complementary strategy: while high-income countries are currently evaluating the impact of passive immunization in infants, maternal immunization offers a cost-effective and globally feasible approach to mitigating the majority of RSV-related morbidity and mortality.

## Data Availability

The datasets used and/or analysed during the current study are available from the corresponding author on reasonable request.

## Abbreviations

RSV: respiratory syncytial virus
